# Evaluation of the Analytical Performance of a High-Precision Immunoassay for the Creatine Kinase-MB Isoenzyme

**DOI:** 10.7759/cureus.96457

**Published:** 2025-11-09

**Authors:** Yukina Kawada, Yoshiyuki Kitamura, Shun-ichiro Asahara, Katsumi Aoyagi, Mitsuhiro Ito, Yoshiaki Kido

**Affiliations:** 1 Research and Development Division, Fujirebio Inc., Tokyo, JPN; 2 Department of Biophysics, Kobe University Graduate School of Health Sciences, Kobe, JPN; 3 Department of Internal Medicine, Kobe University Graduate School of Medicine, Kobe, JPN

**Keywords:** cardiac disease biomarker, chemiluminescent enzyme immunoassay, creatine kinase-mb, diabetes, isoenzyme analysis

## Abstract

Background

Creatine kinase-MB isoenzyme (CK-MB) is an important biomarker for diagnosing myocardial injury. Although cardiac troponin is widely used as the primary biomarker for myocardial infarction, CK-MB measurement remains clinically relevant due to its shorter half-life and utility in detecting reinfarction and estimating infarct size. Here, we established and evaluated a fully automated chemiluminescent enzyme immunoassay (CLEIA; Lumipulse Presto CK-MB) for the LUMIPULSE L2400 analyzer. The primary objective was to assess its analytical performance, and the secondary objective was to explore its potential clinical utility as a cardiac biomarker in patients with diabetes.

Methods

Analytical performance was assessed according to Clinical and Laboratory Standards Institute (CLSI) guidelines, including evaluations of sensitivity, linearity, dilution recovery, precision (repeatability, between-run, between-day, and total), and interference/cross-reactivity. Method comparisons were conducted using a panel of 95 CK-MB-positive serum samples against existing CLEIAs, chemiluminescent immunoassays (CLIA), immunoturbidimetry assays, and a CK-MB activity assay. Pearson correlation and Passing-Bablok regression were used to assess agreement. Discrepant samples were subjected to isoenzyme analysis. An exploratory clinical study measured CK-MB concentrations, as well as multiple other cardiac biomarkers, using banked serum samples from 120 diabetic patients grouped according to cardiovascular disease history and 92 diabetic patients grouped according to hypoglycemic treatment risk.

Results

The assay demonstrated high analytical sensitivity (limit of detection: 0.039 ng/mL; limit of quantification: 0.047 ng/mL), excellent linearity over a range of 0.1-349.0 ng/mL, and robust precision (coefficient of variations (CVs) < 3.5%). Strong correlations were observed with existing CLEIA (r = 1.00, 95% CI: 0.995-0.999), CLIA (r = 0.99, 95% CI: 0.988-0.994), and immunoturbidimetry (r = 1.00, 95% CI: 0.992-0.997) assays for CK-MB quantification. All discordant results were resolved by isoenzyme analysis. CK-MB levels were significantly higher in diabetic patients with a history of myocardial infarction compared with those without cardiovascular disease (p < 0.001).

Conclusions

The CK-MB assay demonstrated excellent sensitivity, precision, and strong correlation with conventional procedures. Isoenzyme analysis provided insights into discrepancies observed with activity-based measurements. Exploratory analysis of diabetic patient samples revealed trends related to cardiovascular history. These findings support the potential utility of this assay in both clinical diagnostics and research settings and encourage further multicentric studies to confirm its clinical usefulness, including exploratory observations suggesting broader applicability in populations at elevated risk for cardiac disease.

## Introduction

Creatine kinase-MB (CK-MB) is an established biomarker used in the diagnosis and risk stratification of patients with chest pain or suspected acute coronary syndrome [1]. Although cardiac troponin (cTn) is currently recommended as the primary biomarker for myocardial infarction diagnosis, major guidelines from the American Heart Association (AHA), the American Association for Clinical Chemistry (AACC), and the Japanese Circulation Society recognize CK-MB mass measurement as the best alternative when cTn assays are unavailable [2,3]. Indeed, CK-MB has a shorter half-life than cTn and has been reported to be more sensitive in detecting reinfarction, as well as helpful in estimating infarct size and predicting mortality [4]. As a consequence, CK-MB measurement remains in use at many medical facilities.

Initial CK-MB activity assays were based on standard biochemical methods. However, enzyme instability and poor preservation of CK-MB activity in blood samples prompted the development of mass assays that quantify both active and inactive CK-MB using biochemical or immunological techniques [5]. Because these two assay types are based on distinct measurement principles, discrepancies in CK-MB results have occasionally been observed, highlighting the importance of comparative evaluation to ensure analytical consistency.

CK is a dimer composed of two subunits, CK-M and CK-B, which combine to form isoenzymes such as CK-MM (predominantly in skeletal and cardiac muscle), CK-MB (mainly in cardiac muscle), and CK-BB (found in the brain and gastrointestinal tract) [6]. In addition, macro-CK complexes are known to exist, including type I (a complex of CK and IgG) and type II (mitochondrial-derived CK, or m-CK). These atypical forms of CK can potentially cause erroneously elevated results in both activity-based and mass-based CK-MB assays, depending on the measurement method and sample characteristics [7].

Diabetes mellitus is a major risk factor for cardiovascular disease. Specifically, it is associated with an increased risk of myocardial infarction and heart failure through mechanisms such as chronic inflammation, atherosclerosis, and diabetic cardiomyopathy [8]. While cardiac biomarkers such as cTn and CK-MB are well established for diagnosing acute cardiac events, there is a paucity of reports on their use for monitoring biomarker fluctuations in chronic cardiac conditions, particularly those involving CK-MB [9]. Furthermore, as cardiovascular risk may vary with antidiabetic treatment regimens [10-12], examining biomarker profiles across therapeutic categories could provide exploratory insights into CK-MB’s broader clinical relevance. Therefore, we included an exploratory component using diabetic patient samples to investigate potential associations between CK-MB levels, cardiovascular history, and antidiabetic medication profiles.

While existing commercial CK-MB assays generally provide acceptable clinical performance, several reports have documented limitations in analytical sensitivity, calibration consistency, and measurement range [13,14]. Therefore, there remains a need to enhance the analytical precision and broaden the dynamic range of CK-MB assays. Such improvements in assay procedures are likely to facilitate more accurate discrimination between patient subgroups in both acute and chronic settings. To achieve these goals, we established and evaluated a fully automated chemiluminescent enzyme immunoassay (CLEIA) for the quantitative measurement of CK-MB (Lumipulse Presto CK-MB), designed for the LUMIPULSE L2400 analyzer, equipped with a short measurement time. The primary objective of this study was to evaluate the analytical performance of the immunoassay, including sensitivity, precision, linearity, and comparison with existing commercial assays. As a secondary objective, we conducted exploratory analyses using serum samples from diabetic patients to assess the potential clinical utility of this assay across cardiovascular histories and treatment backgrounds.

## Materials and methods

Assay principle

Lumipulse Presto CK-MB (Fujirebio, Tokyo, Japan) is an assay kit for the quantitative measurement of CK-MB in serum and plasma samples. It is based on CLEIA technology and employs a two-step sandwich immunoassay designed for the LUMIPULSE L2400 analyzer [15].

In this assay, CK-MB present in the specimen specifically binds to anti-CK-MB monoclonal antibody (mouse) immobilized on ferrite microparticles. CK-MB of the immunocomplexes on the microparticles is detected with an alkaline phosphatase (ALP)-labeled anti-CK-MB monoclonal antibody (mouse). In the first step, a 10 μL aliquot of sample is dispensed into a suspension of anti-CK-MB antibody-coated microparticles and incubated at 37°C for either 8 minutes (normal mode) or 5 minutes (short-time mode). After washing, ALP-labeled anti-CK-MB antibody is added and incubated at 37°C for either 8 minutes (normal mode) or 5 minutes (short-time mode). Following an additional wash step, a substrate solution containing 3-(2'-spiroadamantane)-4-methoxy-4-(3''-phosphoryloxy)phenyl-1,2-dioxetane disodium salt (AMPPD) is added to the reaction mixture. The luminescent signal at 463 nm corresponds to the CK-MB concentration and is generated via AMPPD cleavage. The signal intensity is proportional to the amount of CK-MB present in the sample. The maximum throughput of the LUMIPULSE L2400 instrument is 240 tests per hour using the short-time mode (approximately 15 minutes).

Assay kits

The following commercially available CK-MB assay kits were used for method comparison: Compared kit A: Lumipulse G CK-MB (CLEIA; Fujirebio); compared kit B: ARCHITECT STAT CK-MB (CLIA; Abbott Japan, Tokyo, Japan); compared kit C: L-type Wako CK-MB Mass (latex immunoturbidimetry; Fujifilm Wako, Osaka, Japan); and compared kit D: L-type Wako CK-MB (enzyme activity assay; Fujifilm Wako). Kits A, B, and C are CK-MB mass assays, while kit D is an enzymatic activity-based CK-MB assay.

For the exploratory clinical analysis of serum samples from diabetic patients, three cardiac biomarker assays were used. Troponin I (TnI) was assayed using Lumipulse Presto hs Troponin-I (CLEIA; Fujirebio). B-type natriuretic peptide (BNP) was assayed using Lumipulse Presto BNP (CLEIA; Fujirebio). Myoglobin was assayed using Lumipulse Presto Myoglobin (CLEIA; Fujirebio).

Samples

Clinical samples used for method comparison and exploratory clinical analysis in diabetic patients were obtained from Tsukuba Medical Laboratory of Education and Research Center (Japan) under ethical approval from the Ethics Committee of the University of Tsukuba (Approval No. H29-107). Additional samples, including CK-MB-positive specimens and healthy donor samples, were obtained from Precision for Medicine (formerly ProMedDx, LLC; Norton, MA, USA) and Trina Bioreactives AG (Chicago, IL, USA). Samples were stored at temperatures below -60°C until use. All samples were collected in accordance with ethical standards and relevant regulations and were approved for research use by appropriate institutional review boards (IRBs) or equivalent ethics committees.

Analytical evaluation protocols

All basic analytical performance evaluations, except for the CK isoenzyme identification tests, were performed primarily in accordance with the guidelines of the CLSI.

The Lumipulse Presto CK-MB assay was performed using reagent lots produced during internal manufacturing for analytical performance evaluations and a commercially released lot (F3B5111) for the exploratory clinical analysis using diabetic patient samples. Consistency among reagent lots was confirmed through replicate testing. Calibration was conducted using the Lumipulse Presto CK-MB Calibrator Set (Fujirebio, Tokyo, Japan), and internal quality control materials were analyzed daily to ensure assay stability. The calibration procedure was performed in accordance with the manufacturer’s instructions. Two concentration levels of internal quality control materials (low and high; approximately 50 ng/mL and 150 ng/mL, respectively) were tested each day. The results of the internal quality control materials were verified to be within the acceptable ranges provided by the manufacturer throughout all analytical evaluations. All measurements were performed at room temperature (approximately 20-30°C).

For method comparison, the following commercially available CK-MB assays were evaluated. The Lumipulse G CK-MB assay was performed on the LUMIPULSE G1200 analyzer. The L-type Wako CK-MB Mass and L-type Wako CK-MB (enzyme activity) assays were performed using the 7180 Clinical Analyzer (Hitachi, Tokyo, Japan). Measurements using the ARCHITECT STAT CK-MB assay were outsourced to a clinical laboratory at SRL, Inc. (Tokyo, Japan). The CK isoenzyme identification tests were also outsourced to SRL, Inc. and conducted using the Titan Gel CK assay kit (immunofixation electrophoresis (IFE); HELENA Laboratories Corp., Beaumont, TX, USA).

Sensitivity Tests

The limits of blank (LoB), detection (LoD), and quantitation (LoQ) were determined according to CLSI guideline EP17-A2 [16]. LoB was estimated from four blank samples measured in quintuplicate over three days using a nonparametric 95th percentile method. LoD and LoQ were evaluated using eight low-concentration serum/plasma samples measured in octuplicate over five days. LoD was calculated as LoD = LoB + Cp × SDL, where Cp is the multiplier corresponding to the 95th percentile of the standard normal distribution (typically 1.645), and SDL is the standard deviation of low-concentration samples. LoQ was defined as the concentration corresponding to 10% CV. Normality was assessed using the Shapiro-Wilk test (Analyse-it Ltd., Leeds, UK).

Linearity and Dilution Linearity

Linearity was evaluated in accordance with CLSI guideline EP6-A [17]. Test samples were prepared by mixing low- and high-concentration serum pools in fixed weight ratios ranging from 19:1 to 1:19. The expected values, derived from the mixing ratios and measured concentrations of the two pools, were plotted on the x-axis, while the measured values of the test samples were plotted on the y-axis.

Polynomial regression analysis (first- and third-order) was performed using Analyse-it software, in accordance with CLSI guideline EP6-A, which recommends evaluating linearity by comparing first-, second-, and third-order polynomial fits. Hypothesis testing was conducted using the null hypothesis (H₀: coefficient = 0) and the alternative hypothesis (H₁: b₂ ≠ 0 for the second-order term; b₂ and b₃ ≠ 0 for the third-order terms). If all p-values exceeded 0.05 (i.e., all coefficients were not statistically significant), the test sample range was considered linear.

Dilution linearity was evaluated using five serum and five plasma samples, each diluted with Lumipulse Presto specimen diluent (Fujirebio) at volumetric dilution factors of 10-, 100-, 200-, and 1000-fold. Recovery (%) was calculated as follows:

Recovery (%) = (Measured value of diluted sample × dilution factor) / (Measured value of undiluted sample) × 100

Dilution linearity was considered acceptable when recovery was within 100 ± 15%.

Precision Evaluation

Precision, including repeatability, between-run precision, between-day precision, and total imprecision, was evaluated according to CLSI guideline EP05-A3 [18]. Three serum samples, one plasma sample, and two internal control samples were measured twice per run, twice per day, over 20 days within a 31-day period.

Total imprecision was calculated by combining the variance components from repeatability, between-run, and between-day measurements.

Method Comparison

Method comparison was conducted using a common panel of 95 CK-MB-positive clinical samples. The correlation between the Lumipulse Presto CK-MB assay and four commercial assays (A: Lumipulse G CK-MB; B: ARCHITECT STAT CK-MB; C: L-type Wako CK-MB Mass; D: L-type Wako CK-MB Activity) was evaluated. For each assay, Pearson’s correlation coefficient (r) was calculated using Analyse-it software based on measured values within the reportable range specified in the respective assay instructions, and its 95% confidence interval (95% CI) was estimated using Fisher’s z-transformation. The regression slope, along with its 95% CI, was determined using the Passing-Bablok method with Analyse-it software, also based on values within their reportable range.

The reportable ranges for each assay were as follows: Lumipulse Presto CK-MB and Lumipulse G CK-MB, 1.0-300.0 ng/mL; ARCHITECT STAT CK-MB, 0.1-300.0 ng/mL; L-type Wako CK-MB Mass, 1.0-193.3 ng/mL; and L-type Wako CK-MB (activity assay), 2-2500 U/L.

CK Isoenzyme Identification

CK isoenzyme identification was performed on 20 selected samples, comprising the 10 samples with the smallest differences and the 10 samples with the largest differences between the CK-MB activity assay and the four CK-MB mass assays in the method comparison study. The relative proportions of CK-BB, CK-MB, CK-MM, macro-CK, and mitochondrial CK (m-CK) in each sample were evaluated.

Additional Studies (Authors’ Data Reported in Package Insert)

Additional foundational performance evaluations, including cross-reactivity, interference testing, serum versus plasma correlation, and reference range determination, were performed by the authors during assay development and have been publicly disclosed in the official product package insert (Fujirebio).

Cross-reactivity was assessed against CK-MM (up to 50,000 ng/mL) and CK-BB (up to 1,000 ng/mL) isoenzymes. Interference testing included the evaluation of bilirubin F (18.0 mg/dL), bilirubin C (18.8 mg/dL), hemoglobin (470 mg/dL), chyle (1,590 formazin units), and triglyceride (2,000 mg/dL). Serum and plasma correlation was evaluated using paired samples (n = 70 or 71 per comparison) from individual donors, and the correlation coefficient and regression slope were assessed using the Passing-Bablok method with Analyse-it software. The reference range was established according to CLSI EP28-A3c guidelines using serum samples from 262 apparently healthy subjects [19].

Exploratory clinical analysis using diabetic patient samples

In addition to the analytical performance evaluations, we conducted an exploratory clinical analysis using serum samples from diabetic patients to assess the potential clinical utility of the CK-MB assay. CK-MB values served as the primary measure. To assess consistency across cardiac biomarkers, additional measurements of TnI, BNP, and myoglobin were also conducted. These biomarkers were measured using the LUMIPULSE L2400 analyzer. Serum samples were selected from diabetic patients registered in the biobank based on diagnostic records consistent with ICD-10 codes E10-E14. The selected samples were further stratified into two independent subgroup analyses, as follows:

The first subgroup involved stratification based on cardiovascular disease history. Patients were categorized into three groups based on their cardiovascular history. Group A (No-CVD) had no prior history of cardiovascular disease (i.e., no diagnosis codes under I00-I99). Group B (Non-IHD cardiac history) had a history of non-ischemic heart disease (including I11, I13, I26.0, I27, I30-I52) but no diagnosis of ischemic heart disease (I20-I25). Group C (IHD) had a documented history of ischemic heart disease (I20-I25).

The second subgroup involved stratification based on antidiabetic medication history. From the pool of diabetic samples, a subset was selected after excluding cases with renal disease (ICD-10 codes N00-N29 and N30-N39), primary hypertension (I10-I15), or serum creatinine values outside the reference range (only including samples with creatinine between 0.47 and 1.04 mg/dL). These samples were divided into the following three medication-related groups. Group D (no medication) had no history of antidiabetic medication. Group E (low risk of hypoglycemia) had taken DPP-4 inhibitors or SGLT2 inhibitors (code 3969) or biguanides (code 3962) but no insulin or sulfonylureas. Group F (high risk of hypoglycemia) had a history of insulin (code 2492) or sulfonylurea (code 3961) use. Of the 43 samples classified into Group F, 32 were also concurrently prescribed medications corresponding to Group E.

Inclusion and exclusion criteria

Serum samples were eligible if obtained from patients with a documented diagnosis of diabetes mellitus (ICD-10 codes E10-E14) and registered in the institutional biobank with available diagnostic and prescription records. To avoid duplication, only one specimen per patient was included. Samples were excluded if essential diagnostic or prescription information was missing or incomplete.

For the subgroup analysis based on antidiabetic medication use, additional exclusion criteria were applied. Specifically, samples from patients with renal disease (ICD-10 codes N00-N39), primary hypertension (I10-I15), or serum creatinine values outside the reference range (0.47-1.04 mg/dL) were excluded.

Eligible samples were selected primarily through random extraction from the biobank. This random selection was performed using an automated, computer-based procedure to avoid human bias. In rare cases where multiple specimens from the same patient fulfilled all criteria, the sample with the highest available troponin T value was preferentially selected, based on prior evidence that peak troponin levels may better reflect underlying cardiovascular burden [20-22]. When troponin T data were unavailable, random selection was applied. The exact timing of blood collection relative to disease onset or medication initiation was not available.

Statistical analysis

All statistical analyses were performed using EZR software (version 4.3.1; Saitama Medical Center, Jichi Medical University, Japan), a graphical user interface for R [23]. A two-step analysis was conducted for each comparison. Descriptive statistics were summarized for each group, with age and sex aggregated by group. The distribution of continuous variables was assessed using the Shapiro-Wilk test, which indicated non-normal distributions. Therefore, continuous variables were reported as medians and IQRs, and group comparisons were performed using the Kruskal-Wallis test. Categorical variables were compared using the chi-square test or Fisher’s exact test, as appropriate.

To adjust for potential confounding factors, particularly age and sex, linear regression analysis was conducted to evaluate whether group differences in biomarker levels were independent of these covariates. A p-value < 0.05 was considered statistically significant. The exploratory nature of this study and the sample sizes in each subgroup (Groups A-C: 40 each; Groups D-F: 32, 17, and 43, respectively) should be noted. Multivariate analyses were conducted to adjust for age and sex, acknowledging that the sample sizes may limit the ability to detect moderate differences between groups [24].

## Results

Analytical evaluation

Sensitivity

**Figure 1:**
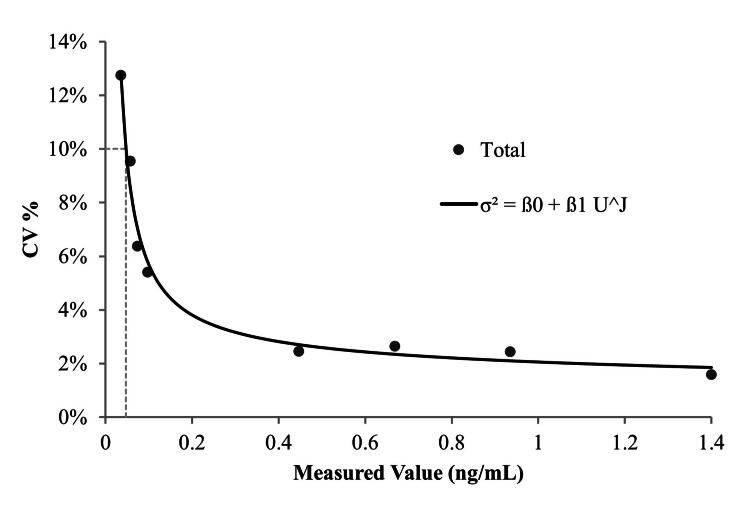
Determination of the limit of quantitation (LoQ). The coefficient of variation (CV) values were plotted against the measured concentrations of low-level serum/plasma samples. The LoQ was defined as the lowest concentration at which the CV was ≤10%, in accordance with CLSI EP17-A2 guidelines. Each point represents the mean CV obtained from octuplicate measurements conducted over five days. The horizontal dashed line indicates the 10% CV threshold. CLSI: Clinical and Laboratory Standards Institute.

The LoB was determined to be 0.007 ng/mL using the nonparametric method in accordance with CLSI guideline EP17-A2. The LoD was subsequently calculated as follows: LoB (0.007 ng/mL) + Cp (1.646) × SDL (0.018 ng/mL) = 0.037 ng/mL. The LoQ was estimated to be 0.047 ng/mL, corresponding to a CK-MB concentration with 10% imprecision (Figure 1).

Linearity

**Figure 2:**
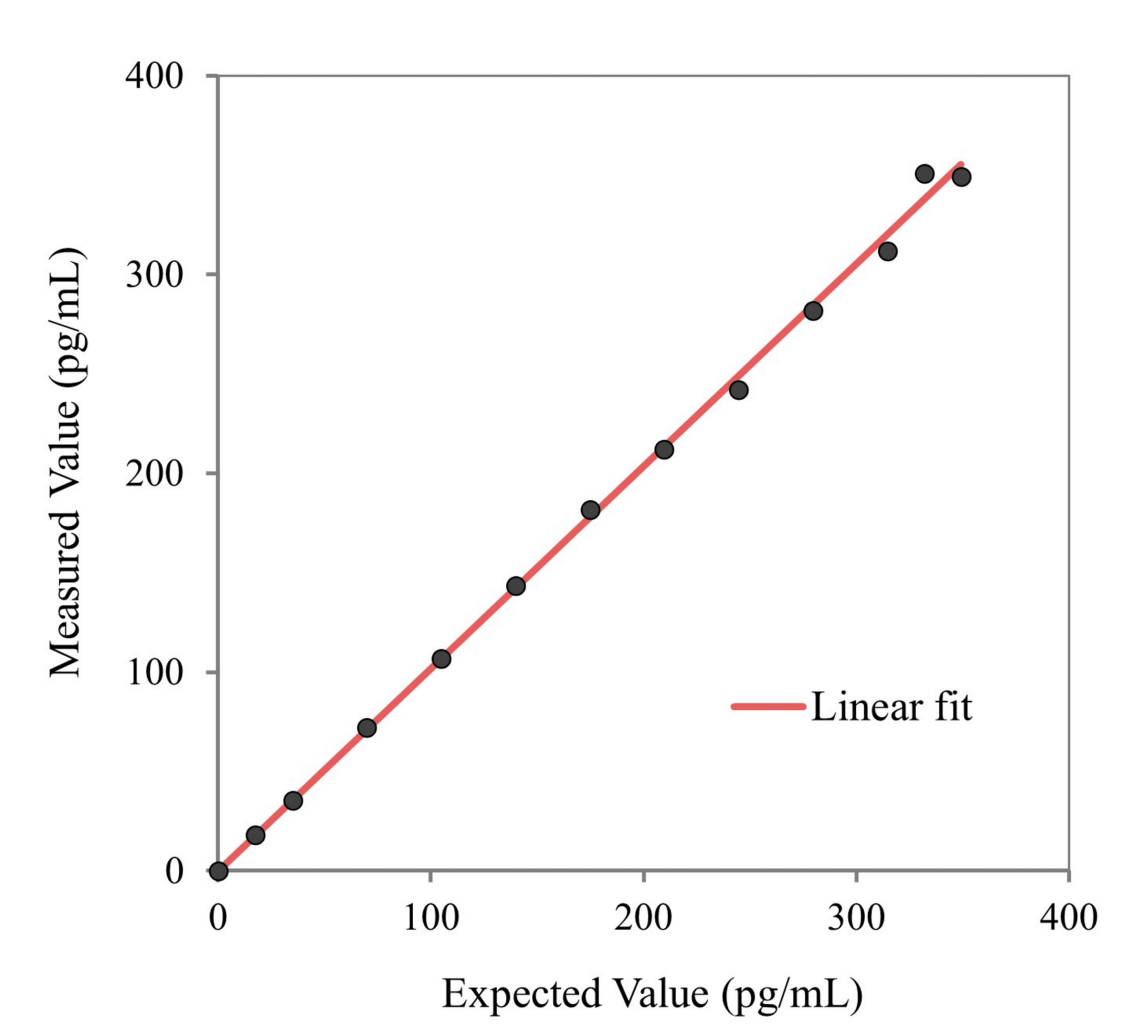
Linearity assessment using polynomial regression analysis. Expected concentrations (based on mixing ratios of low- and high-concentration serum pools) are plotted on the x-axis, and measured concentrations are plotted on the y-axis. Polynomial regression analysis (first-, second-, and third-order) was performed to evaluate linearity in accordance with CLSI EP6-A guidelines. All p-values for higher-order terms exceeded 0.05, indicating no significant deviation from linearity across the tested range (0.1-349.0 ng/mL). CLSI: Clinical and Laboratory Standards Institute.

A scatter plot was drawn with the expected values on the x-axis and the measured values on the y-axis (Figure 2). Polynomial regression analysis showed that the p-values for the quadratic term (b₂) in the quadratic and cubic models were 0.141 and 0.494, respectively, and the p-value for the cubic term (b₃) in the cubic model was 0.724 (Tables 1-2). As all p-values exceeded 0.05, no statistically significant deviation from linearity was observed, confirming linearity over the concentration range of 0.1-349.0 ng/mL.

**Table 1:** Polynomial regression analysis for linearity assessment.

Statistical Hypothesis Testing		b₀	b₁	b₂	b₃
Linear regression	Coefficient	-0.002	1.02E+00	-	-
Quadratic regression	Coefficient	-0.003	1.03E+00	-4.92E-05	-
	p-value	-	-	0.141	-
Cubic regression	Coefficient	-0.003	1.03E+00	-9.83E-05	1.33E-07
	p-value	-	-	0.494	0.724

**Table 2:** Comparison of expected and measured concentrations in linearity testing.

Test Sample	Average Measured Value (ng/mL)	Expected Value (ng/mL)	Measured / Expected (%)
1	0.1	0.1	100
2	18	17.6	103
3	35.6	35	102
4	72.1	69.9	103
5	106.9	104.9	102
6	143.3	139.7	103
7	181.6	174.6	104
8	212	209.6	101
9	241.9	244.7	99
10	281.9	279.6	101
11	311.6	314.4	99
12	350.6	331.8	106
13	349	349	100

Dilution Linearity

The recovery rate for five serum test samples and five plasma samples ranged from 90% to 107% up to 1000-fold dilution, remaining within the acceptable range of 100 ± 15% (Table 3).

**Table 3:** Recovery rates in dilution linearity testing of serum and plasma samples. EDTA: Ethylenediaminetetraacetic acid.

Test Sample	1	2	3	4	5	6	7	8	9	10
Sample type	Serum	Serum	Serum	Serum	Serum	EDTA-2Na Plasma	EDTA-K₂ Plasma	Na Heparin Plasma	Na Heparin Plasma	Na Citrate Plasma
Measured value (ng/mL)										
Undiluted	1067.4	1058.9	1130.8	1133.7	1121.6	1041.3	1119	1142.7	1138.6	1119.3
10-fold dilution	106.5	106.5	113.6	114.4	115.1	107.8	118.5	122.6	120.8	117.7
100-fold dilution	10.5	10.4	11.3	11.2	11.1	10.7	11.5	11.7	11.9	11.6
200-fold dilution	5.1	4.9	5.4	5.5	5.3	5.2	5.8	5.8	5.8	5.6
1000-fold dilution	1	1	1.1	1	1.1	1.1	1.1	1.2	1.2	1.1
Recovery rate (%)										
10-fold dilution	100	101	100	101	103	103	106	107	106	105
100-fold dilution	98	98	100	99	99	103	103	102	104	103
200-fold dilution	96	92	96	96	95	100	103	102	102	100
1000-fold dilution	93	94	96	90	99	101	102	101	101	101

Precision Verification

Repeatability, between-run reproducibility, between-day reproducibility, and total imprecision are shown in Table 4. The %CVs were 0.9-1.2%, 1.1-2.0%, 2.0-3.0%, and 2.7-3.5%, respectively. All values were within the acceptable range (<10.0%). The concentration levels of the tested samples are also presented in Table 4 as the mean measured values, representing low to high CK-MB concentrations within the analytical measurement range.

**Table 4:** Precision evaluation of serum, plasma, and internal control samples. N: Number of measurements (sample size); CV: Coefficient of Variation; Serum L: Serum sample (Low concentration); Serum M: Serum sample (Medium concentration); Serum H: Serum sample (High concentration); Plasma M: Plasma sample (Medium concentration).

	N	Mean measured value (ng/mL)	Repeatability (CV%)	Between-run CV (%)	Between-day CV (%)	Total CV (%)	95% CI (Lower limit)	95% CI (Upper limit)
Quality control materials								
1	80	50.2	1	2	2.5	3.4	2.7	4.5
2	80	143.4	1	1.5	2	2.7	2.2	3.5
Test samples								
Serum L	80	19.3	1.1	1.1	2.3	2.8	2.2	3.8
Serum M	80	101.8	1.2	1.6	2.2	3	2.4	4
Serum H	80	175.1	0.9	1.2	2.4	2.8	2.2	3.8
Plasma M	80	89.9	1.2	1.4	3	3.5	2.8	4.8

Method Comparison

**Figure 3:**
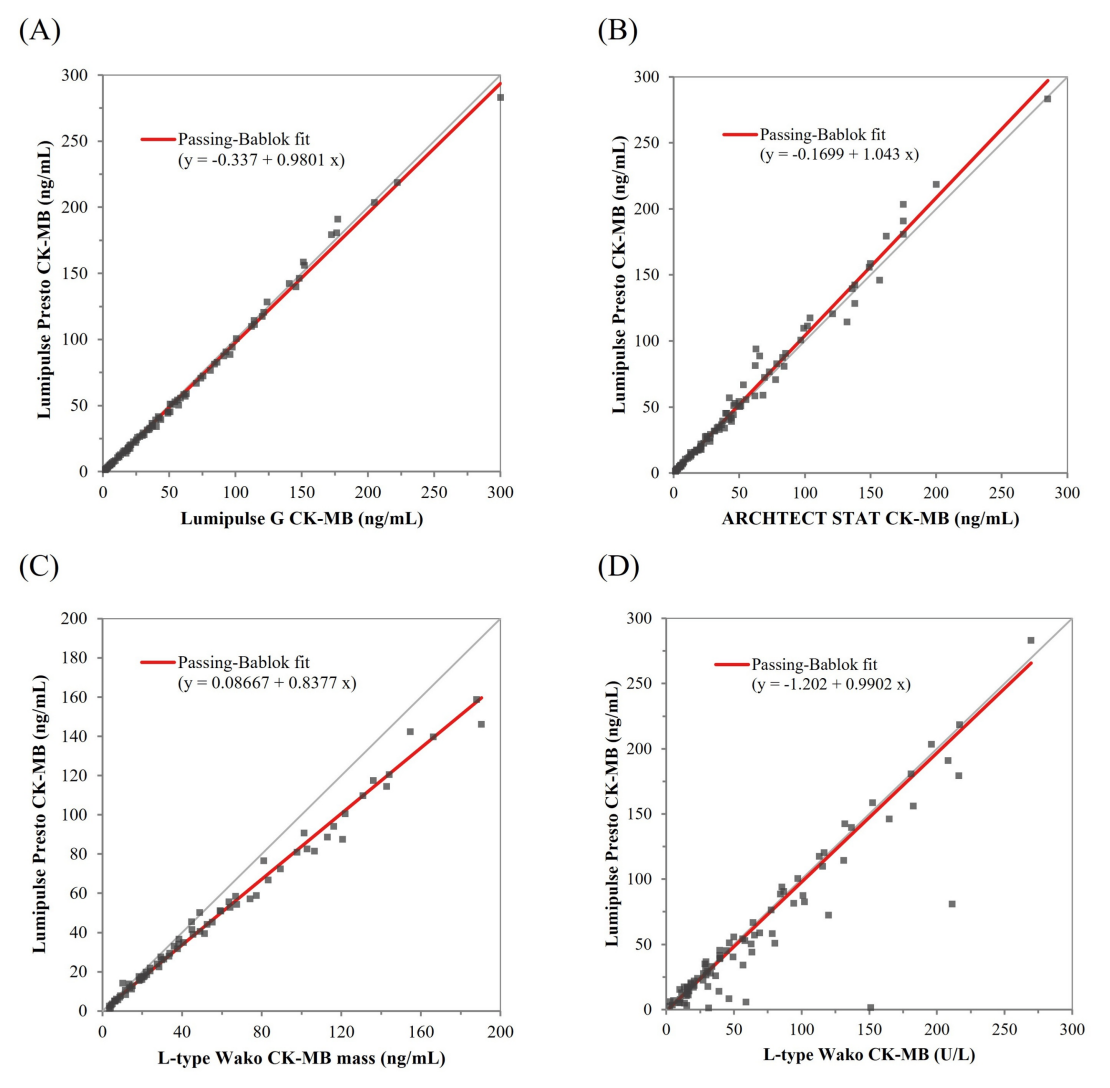
Method comparison between Lumipulse Presto CK-MB and four commercial CK-MB assays. A scatter plot showing the comparison between the Lumipulse Presto CK-MB assay (y-axis) and four commercial CK-MB assays (x-axis): (A) Lumipulse G CK-MB, (B) ARCHITECT STAT CK-MB, (C) L-type Wako CK-MB Mass, and (D) L-type Wako CK-MB Activity. Correlation coefficients and regression slopes were calculated using the Passing-Bablok method. CK-MB: Creatine kinase-MB.

A scatter diagram was drawn with the measured values of the compared kit on the x-axis and those of the Lumipulse Presto CK-MB on the y-axis (Figure 3). The correlation coefficient and regression slope were 1.00 (95% CI: 0.995-0.999) and 0.98 (95% CI: 0.96-0.99) against the Lumipulse G CK-MB assay; 0.99 (95% CI: 0.988-0.994) and 1.04 (95% CI: 1.01-1.07) against the ARCHITECT STAT CK-MB assay; 1.00 (95% CI: 0.992-0.997) and 0.84 (95% CI: 0.81-0.86) against the L-type Wako CK-MB mass assay; and 0.92 (95% CI: 0.879-0.948) and 0.99 (95% CI: 0.90-1.04) against the L-type Wako CK-MB activity assay.

CK Isoenzyme Identification Test

The content ratios of CK-BB, CK-MB, CK-MM, macro-CK, and m-CK for each specimen are shown in Table 5. Six out of 10 specimens that showed larger differences in measured values between the L-type Wako CK-MB assay kits and the other four CK-MB mass assay kits exhibited a relative increase in the content of macro-CK, m-CK, or CK-BB isoenzyme. In contrast, three out of 10 specimens that showed the least difference in measured values during method comparison displayed a relative increase in the content of macro-CK.

**Table 5:** Creatine kinase (CK) isoenzyme composition in specimens with assay discrepancies.

	Quantitative Assay				Enzyme Activity Assay	Variability between Enzyme Activity Assay and Lumipulse Presto CK-MB	CK Isoenzyme Composition (%)				
Test Kit	Lumipulse Presto CK-MB	Lumipulse G CK-MB	ARCHITECT STAT CK-MB	L-type Wako CK-MB Mass	L-type Wako CK-MB		CK-BB	CK-MB	CK-MM	Macro-CK	m-CK
Unit of measure	ng/mL	ng/mL	ng/mL	ng/mL	U/L	(%)	(%)	(%)	(%)	(%)	(%)
Discordant samples											
1	27.5	29.7	24.1	29.3	27.5	100	1	12	82	5	-
2	33	35.1	34.8	36	33.6	102	0	17	83	-	-
3	39.5	43.7	37.4	51.2	39.8	101	0	20	66	14	-
4	39	39.6	44.2	45.4	39.4	101	0	16	84	-	-
5	45.3	49.2	39.7	55.2	44.5	98	0	16	72	12	-
6	76.5	81.1	72.8	81	77.4	101	1	15	84	-	-
7	120.5	121.2	121	144.1	116.7	97	0	12	88	-	-
8	139.7	145.7	136	166.1	136.9	98	0	17	83	-	-
9	180.7	176.2	175	Range over	180.9	100	0	7	93	-	-
10	218.6	222.1	200	Range over	216.7	99	1	31	68	-	-
Concordant samples											
11	72.4	75.5	69.1	89.3	120	166	1	14	85	-	-
12	80.9	120.1	84.1	97.7	211.3	261	1	7	75	17	-
13	4.9	5.3	4.4	6.1	13.6	278	0	2	98	-	-
14	13.9	17.3	13.3	13.3	38.8	279	0	1	99	-	-
15	3.1	3.3	2.9	4.3	15	484	2	4	94	-	-
16	8.4	9.3	7.6	11.7	46.3	551	3	6	84	3	4
17	5.7	6.7	5.1	7.7	58.8	1032	0	24	66	10	-
18	1.3	1.6	1.4	3.4	31.2	2400	34	5	61	-	-
19	0.9	1.2	0.9	1	36.4	4044	31	9	60	-	-
20	1.5	1.6	1.3	4	151	10067	1	5	40	54	-

Additional Studies (Authors’ Data Reported in Package Insert)

Cross-reactivity of the assay was evaluated against CK-MM and CK-BB isoenzymes. No significant cross-reactivity was observed up to 50,000 ng/mL of CK-MM or 1,000 ng/mL of CK-BB. The effects of potential interfering substances, including bilirubin F (up to 18.0 mg/dL), bilirubin C (up to 18.8 mg/dL), hemoglobin (up to 470 mg/dL), chyle (up to 1,590 FTU), and triglyceride (up to 2,000 mg/dL), were tested. No significant interference was observed under the test conditions.

The correlation between serum and various plasma sample types (including lithium heparin, sodium heparin, EDTA-2K, EDTA-2Na, and sodium citrate plasma) was evaluated using 70 or 71 paired samples for each comparison. The correlation coefficient (R) was 1.00, and the slope ranged from 0.99 to 1.01, indicating excellent agreement.

The upper reference limits (URLs) of serum CK-MB concentrations were determined using a nonparametric approach based on samples from 262 apparently healthy individuals (130 males and 132 females). In the overall population, the 99th and 97.5th percentile URLs were 3.9 ng/mL and 3.7 ng/mL, respectively. In males, the corresponding values were 4.5 ng/mL and 5.1 ng/mL, while in females they were 2.7 ng/mL and 2.4 ng/mL, respectively.

Exploratory clinical analysis using diabetic patient samples

Comparison by Cardiovascular History (Groups A-C)

**Figure 4:**
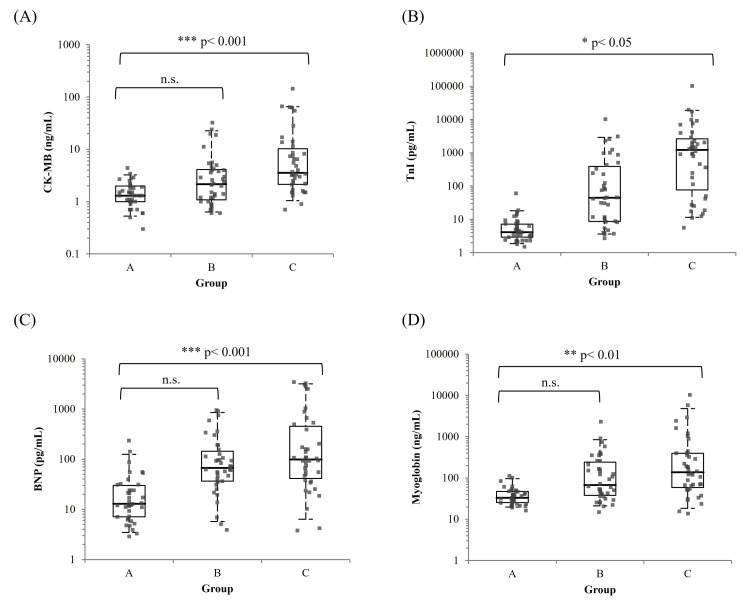
Box plots of serum CK-MB, TnI, BNP, and myoglobin levels across Groups A (No-CVD), B (Non-IHD), and C (IHD). The boxes represent the IQR; the horizontal line within each box indicates the median, and whiskers extend from the 5th to the 95th percentiles. The y-axis is plotted on a logarithmic scale. Subfigures A, B, C, and D correspond to CK-MB, TnI, BNP, and myoglobin, respectively. *p < 0.05; **p < 0.01; ***p < 0.001; n.s., not statistically significant. CK-MB: Creatine Kinase-MB Isoenzyme; TnI: Troponin I; BNP: B-Type Natriuretic Peptide; CVD: Cardiovascular Disease; IHD: Ischemic Heart Disease; n.s.: Not Statistically Significant.

A total of 120 diabetic patients were analyzed, equally divided into three groups according to their cardiovascular history: Group A (No-CVD), Group B (Non-IHD cardiac history), and Group C (IHD). Age and sex distributions were comparable across the three groups (Table 6). Serum CK-MB levels showed a significant stepwise increase from Group A to Group C, with median values of 1.3, 2.15, and 3.55 ng/mL, respectively (p < 0.001). Similar increasing trends were observed for TnI (4.1, 44.7, and 1209.4 pg/mL), BNP (12.9, 66.9, and 99.1 pg/mL), and myoglobin (33.0, 67.2, and 137.2 ng/mL), all with p < 0.001. These trends are visually evident in the box plots of each biomarker (Figure 4). To control for age and sex as potential confounders, multivariate linear regression was performed. After adjustment, CK-MB remained significantly elevated in Group C compared with Group A (β = 12.77, 95% CI: 5.42-20.11, p < 0.001), as did TnI (β = 5026.5, p = 0.020), BNP (β = 505.4, p < 0.001), and myoglobin (β = 748.4, p = 0.004) (Table 7). No statistically significant differences were observed between Group A and Group B for any biomarker after adjustment.

**Table 6:** Descriptive statistics of cardiac biomarkers and demographic characteristics across Groups A-C. CVD: Cardiovascular Disease; IHD: Ischemic Heart Disease; CK-MB: Creatine Kinase-MB Isoenzyme; TnI: Troponin I; BNP: B-Type Natriuretic Peptide.

Variable	Group A (No-CVD)	Group B (Non-IHD)	Group C (IHD)	p-value
n	40	40	40	-
Male (%)	30 (75%)	30 (75%)	30 (75%)	1
Age (years)	73.0 (64.0-79.0)	75.5 (60.0-84.0)	75.5 (61.0-90.0)	0.152
CK-MB (ng/mL)	1.3 (0.30-4.40)	2.15 (0.60-32.30)	3.55 (0.70-145.00)	<0.001
TnI (pg/mL)	4.1 (1.50-60.50)	44.7 (2.70-10,269.40)	1,209.4 (5.60-101,107.00)	<0.001
BNP (pg/mL)	12.9 (2.90-234.40)	66.9 (3.90-954.50)	99.1 (3.80-3,449.10)	<0.001
Myoglobin (ng/mL)	33.0 (16.20-112.70)	67.2 (14.90-2,309.90)	137.2 (13.70-10,244.10)	<0.001

**Table 7:** Results of linear regression for each biomarker across Groups A-C (adjusted for age and sex). CK-MB: Creatine Kinase-MB Isoenzyme; TnI: Troponin I; BNP: B-Type Natriuretic Peptide.

Variable	Group B vs A (β (95% CI), p)	Group C vs A (β (95% CI), p)
CK-MB	2.93 (-4.37, 10.23), p = 0.428	12.77 (5.42, 20.11), p < 0.001
TnI	550.6 (-3,642.1, 4,743.2), p = 0.795	5,026.5 (808.4, 9,244.5), p = 0.020
BNP	133.1 (-128.5, 394.7), p = 0.316	505.4 (242.2, 768.6), p < 0.001
Myoglobin	191.9 (-307.0, 690.8), p = 0.448	748.4 (246.5, 1,250.3), p = 0.004

Subgroup Analysis by Antidiabetic Medication History (Groups D-F)

**Figure 5:**
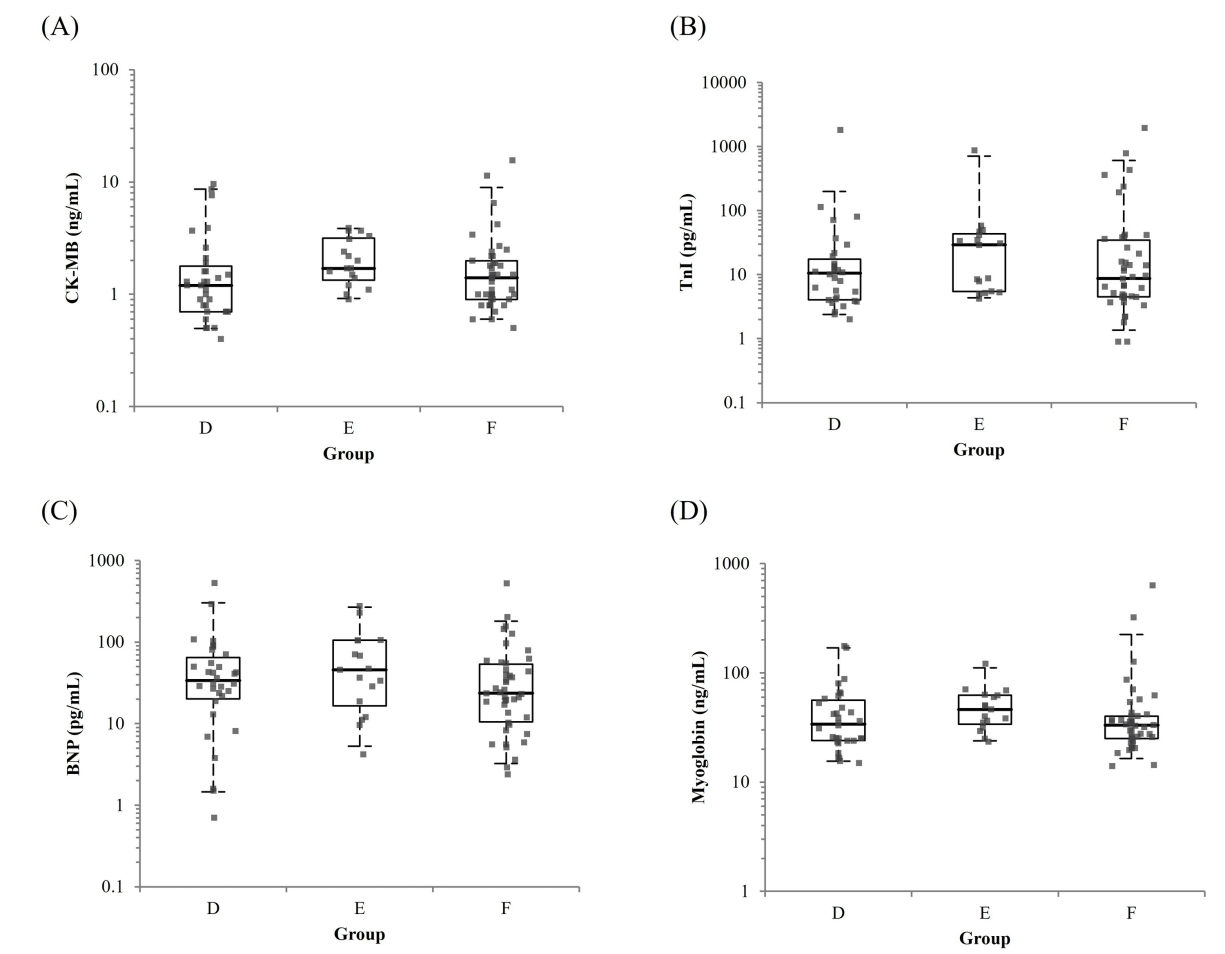
Box plots of serum CK-MB, TnI, BNP, and myoglobin levels across Groups D (No medication), E (Low risk of hypoglycemia), and F (High risk of hypoglycemia). The boxes represent the IQR; the horizontal line within each box indicates the median, and whiskers extend from the 5th to the 95th percentiles. The y-axis is plotted on a logarithmic scale. Subfigures A, B, C, and D correspond to CK-MB, TnI, BNP, and myoglobin, respectively. CK-MB: Creatine Kinase-MB Isoenzyme; TnI: Troponin I; BNP: B-Type Natriuretic Peptide.

A total of 92 diabetic patients were analyzed and categorized into three groups based on antidiabetic medication history: Group D (no medication), Group E (low hypoglycemia risk), and Group F (high hypoglycemia risk). All patients included in this analysis had normal serum creatinine levels and no history of renal disease or primary hypertension. Box plots illustrating the distributions of each cardiac biomarker are presented in Figure 5, with corresponding descriptive and regression results shown in Tables 8-9. No consistent trend in biomarker levels was observed across the three medication-related groups. While CK-MB levels differed significantly in the unadjusted analysis (p = 0.039), the group with higher hypoglycemia risk (Group F) did not show higher values. After adjustment for age and sex in the multivariate regression analysis, no statistically significant differences were observed among Groups D, E, and F for any of the biomarkers.

**Table 8:** Descriptive statistics of cardiac biomarkers and demographic characteristics across Groups D-F. CK-MB: Creatine Kinase-MB Isoenzyme; TnI: Troponin I; BNP: B-Type Natriuretic Peptide.

Variable	Group D (No medication)	Group E (Low risk of hypoglycemia)	Group F (High risk of hypoglycemia)	p-value
n	32	17	43	-
Male (%)	25 (78.1%)	11 (64.7%)	23 (53.5%)	0.089
Age (years)	75.0 (34.0-89.0)	71.0 (56.0-98.0)	74.0 (53.0-83.0)	0.741
CK-MB (ng/mL)	1.2 (0.40-9.60)	1.7 (0.90-3.90)	1.4 (0.50-15.60)	0.039
TnI (pg/mL)	10.5 (2.00-1,809.70)	29.2 (4.20-872.50)	8.7 (0.90-1,951.90)	0.292
BNP (pg/mL)	33.85 (0.70-531.90)	45.8 (4.20-277.60)	23.6 (2.40-527.90)	0.234
Myoglobin (ng/mL)	34.0 (14.90-176.00)	46.3 (23.50-121.00)	33.3 (14.00-635.00)	0.092

**Table 9:** Results of linear regression for each biomarker across Groups D-F (adjusted for age and sex). CK-MB: Creatine Kinase-MB Isoenzyme; TnI: Troponin I; BNP: B-Type Natriuretic Peptide.

Variable	Group E vs D (β (95% CI), p)	Group F vs D (β (95% CI), p)
CK-MB	0.12 (-1.33, 1.57), p = 0.869	0.11 (-1.04, 1.26), p = 0.852
TnI	10.2 (-172.3, 192.6), p = 0.912	49.3 (-95.9, 194.5), p = 0.502
BNP	2.71 (-49.69, 55.11), p = 0.918	-19.91 (-61.41, 22.00), p = 0.350
Myoglobin	5.51 (-38.93, 49.95), p = 0.806	14.46 (-20.91, 49.83), p = 0.419

## Discussion

In this study, we conducted a basic performance evaluation of the Lumipulse Presto CK-MB assay in accordance with CLSI guidelines [16-19]. The results demonstrated excellent analytical sensitivity, with the LoB, LoD, and LoQ determined to be 0.007 ng/mL, 0.037 ng/mL, and 0.047 ng/mL, respectively. These values were markedly lower than the lower quantification limits reported for comparable commercial immunoassay kits (e.g., LoD = 0.3 ng/mL for Elecsys CK-MBII, LoQ = 0.29 ng/mL for ARCHITECT STAT CK-MB, and lower limit of measurement range = 1 ng/mL for L-type Wako CK-MB mass [25-27]), indicating superior sensitivity performance.

The assay also exhibited very high precision, with total precision CVs ≤ 3.5%. In addition to demonstrating a broad clinical linearity range of 0.1-349 ng/mL, the assay maintained dilution linearity up to 1,000-fold, enabling reliable reporting of final values even for highly concentrated samples, surpassing the performance of some other commercial assays [28]. Notably, the required sample volume was only 10 μL, which is lower than or comparable to other platforms (ARCHITECT STAT CK-MB: 80 μL; Elecsys CK-MBII: 15 μL; L-type Wako CK-MB mass: 10 μL), representing an advantage in testing efficiency and sample conservation.

Correlation analysis showed strong agreement with all comparator CK-MB mass assays (Lumipulse G CK-MB, ARCHITECT STAT CK-MB, and L-type Wako CK-MB mass) as well as with the CK-MB activity assay (L-type Wako CK-MB). While regression slopes were favorable for comparisons with other CK-MB mass immunoassays, values measured with the L-type Wako CK-MB mass (latex immunoturbidimetric method) were slightly lower.

In CK isoenzyme identification testing, samples showing discrepancies between CK-MB activity and CK-MB mass assays exhibited elevated proportions of macro-CK, mitochondrial CK, and CK-BB. This finding is consistent with previous reports and suggests potential cross-reactivity of CK-BB and false-positive elevations due to macro-CK and mitochondrial CK in biochemical measurement methods [29,30]. Because the presence of these isoenzymes does not reflect acute myocardial infarction, CK-MB mass immunoassays, which are less affected by this interference, may be better suited for emergency cardiac testing. In particular, patients with conditions associated with macro-CK or CK-BB elevation, such as autoimmune diseases, malignancies, or hepatic disorders, may show falsely elevated CK-MB activity results [31]. Therefore, caution is warranted when interpreting CK-MB activity in such populations, whereas mass assays provide more reliable results under these circumstances.

The correlation between CK-MB mass and CK-MB activity was high, with an estimated unit conversion factor (slope) of approximately 0.99. Previous studies have reported conversion factors of 0.94 or 0.97. However, it should be noted that the clearance rates of active and inactive CK-MB differ over time after myocardial infarction [32]. This variability suggests that conversion between CK-MB activity and mass values cannot always be achieved using a simple constant. Therefore, when transitioning from CK-MB activity assays to CK-MB mass assays in clinical practice, careful consideration should be given to maintaining the continuity of individual patient results.

These analytical performance results demonstrate that the Lumipulse Presto CK-MB kit offers superior sensitivity, precision, and linearity compared with other commercially available immunoassay kits. Such robust characteristics suggest that the Lumipulse Presto CK-MB kit can provide highly reliable measurements even with minimal sample volume, which is particularly advantageous in clinical settings where sample availability is limited. To further explore its potential clinical utility, we conducted an exploratory analysis using serum samples from diabetic patients, aiming to assess the consistency of CK-MB measurements in relation to other cardiac biomarkers across different cardiovascular backgrounds.

Diabetes mellitus is widely recognized as a high-risk condition for myocardial infarction through multiple pathways, including accelerated atherosclerosis, microvascular dysfunction, and chronic low-grade inflammation. In addition, recent reports have described a condition known as diabetic cardiomyopathy, a form of myocardial dysfunction specific to diabetic patients, characterized by chronic inflammation and metabolic alterations independent of hypertension- or ischemia-induced cardiomyopathy [8].

In the present exploratory analysis, the use of biobank samples imposed certain limitations, particularly the lack of information regarding the temporal relationship between cardiovascular events and blood sampling. While the analytical validation was conducted in accordance with CLSI guidelines using an adequate number of samples for each evaluation item, the exploratory clinical analysis should be interpreted as preliminary due to the limited cohort size and retrospective nature of the biobank data. Although multivariate linear regression was applied to adjust for age and sex, the modest sample size in each subgroup limits the statistical power to detect moderate differences in biomarker levels. In addition, potential biological variability and unmeasured confounding factors, such as disease duration, glycemic control, or comorbidities, may have influenced biomarker levels in the diabetic cohort and could not be fully accounted for in this analysis.

Nevertheless, consistent with guideline recommendations, patients with a documented history of myocardial infarction (Group C) showed significantly higher CK-MB levels compared with those without cardiovascular disease (Group A). Similar trends were also observed for the other three cardiac biomarkers (TnI, BNP, and myoglobin). By contrast, patients with non-ischemic cardiac histories (Group B) did not show statistically significant differences compared with Group A. However, median values and overall distributions suggested a tendency toward higher biomarker levels in Group B, which may indicate subclinical or chronic myocardial injury [33]. The absence of statistical significance may be explained, at least in part, by the possibility that samples were not collected during periods of active disease. Nonetheless, an upward trend was still observed despite these limitations. These observations suggest that further studies, particularly those using samples obtained during active disease, could reveal potential clinical relevance even in non-ischemic cardiac conditions. In this context, the combined use of multiple cardiac biomarkers may enhance risk detection by compensating for individual assay limitations.

Regarding antidiabetic therapy, sulfonylureas and insulin have been associated in previous studies with an increased risk of myocardial injury due to hypoglycemia. By contrast, therapeutic agents with a low hypoglycemia risk, such as DPP-4 inhibitors and SGLT2 inhibitors, are linked to lower cardiovascular event rates. Indeed, there is increasing evidence that SGLT2 inhibitors can reduce heart failure readmissions and cardiovascular mortality [11,34]. In the present study, however, no clinically meaningful differences in cardiac biomarker levels were detected between groups stratified by the hypoglycemia risk of their antidiabetic medications. While this might appear inconsistent with prior literature, several factors could account for the findings. Disease severity, diabetes duration, and glycemic control status were unknown. Moreover, many patients in the high-risk group (insulin or sulfonylurea users; Group F) were also receiving agents from the low-risk category (e.g., DPP-4 inhibitors, SGLT2 inhibitors, or biguanides). Specifically, 32 of 43 samples in Group F were from patients receiving such co-medications. This likely reflects real-world clinical practice, where combination therapy is common in patients requiring stricter glycemic control. Such regimens may have mitigated cardiovascular risk in some patients, obscuring group differences. Thus, the present analysis should be regarded as an initial exploratory study. Nonetheless, these findings suggest that future prospective studies tracking cardiovascular events are warranted to clarify biomarker-drug associations while accounting for treatment combinations.

Overall, Lumipulse Presto CK-MB demonstrated biomarker trends consistent with TnI and other established cardiac markers, supporting its potential clinical utility in diagnosing myocardial infarction as per current guidelines [2]. While cardiac troponin is widely used as the primary biomarker for the diagnosis of acute myocardial infarction, CK-MB retains clinical value in specific contexts. In particular, an assay with enhanced sensitivity, precision, and measurement range, such as the Lumipulse Presto CK-MB, may serve as a practical option in facilities without cTn testing. Such testing could be performed for the detection of reinfarction or as part of a multi-marker strategy to broaden cardiovascular risk assessment. This complementary role could be especially relevant in chronic or non-acute cardiac conditions, where combining CK-MB with other markers such as TnI or BNP may improve the detection of subclinical myocardial injury. Furthermore, the assay requires only 10 μL of serum, which is considerably less than typical volumes for TnI (50-100 μL), BNP (50-100 μL), or myoglobin (50-100 μL). The smaller sample size offers the advantage of reducing patient burden during follow-up testing. In addition, under the Japanese reimbursement system, CK-MB testing incurs slightly lower procedural costs (90 points) compared with TnI (109 points), BNP (130 points), and myoglobin (131 points), while sharing the same interpretation fee (144 points). These features make CK-MB an attractive choice for routine use or as part of a multi-marker panel in clinical practice.

## Conclusions

The Lumipulse Presto CK-MB assay demonstrated excellent analytical performance, meeting clinical diagnostic requirements with improved sensitivity, a broad dynamic range, and high reproducibility compared with existing assays. The assay requires only a minimal sample volume and provides rapid, reliable measurements consistent with major cardiac biomarkers, thereby facilitating early diagnosis and risk stratification in acute coronary syndromes.

While the clinical observations in this exploratory study were limited in scale, they suggest that CK-MB may provide additional information for monitoring chronic or subclinical myocardial injury, in addition to its established role in the diagnosis of acute coronary syndromes, with potential relevance for high-risk populations such as diabetic patients. Further multicentric studies are encouraged to confirm these findings and to explore the assay’s clinical relevance, particularly in populations at elevated risk for cardiac diseases.
